# Doppler-encoded Mie scattering rainbow of flying particles

**DOI:** 10.1126/sciadv.aef7659

**Published:** 2026-06-17

**Authors:** Rui Wang, Pushihan Wang, Yuxuan Lang, Yao Zhang, Chenjie Liang, Shangran Xie

**Affiliations:** ^1^ School of Optics and Photonics, Beijing Institute of Technology, Beijing 100081, China.; ^2^ Key Laboratory of Photonic Information Technology, Ministry of Industry and Information Technology, Beijing 100081, China.; ^3^ National Key Laboratory on Near-Surface Detection, Beijing 100072, China.

## Abstract

Light scattering constitutes the most fundamental process in light-matter interactions and serves as the cornerstone of modern particle metrology. Although numerous scattering-based techniques have been established for characterizing particles in constrained motion, the precise analysis of freely flying particles remains largely underdeveloped. This limitation stems primarily from the severe constraints on accessible data acquisition time and the motion-induced image blur inherent in conventional scattering detection schemes. Here, we report the observation of a Doppler-encoded Mie scattering effect using optically propelled microparticles in antiresonant hollow-core fibers and further introduce a transverse Doppler spectrometry for single-particle-level metrology of flying particles. It is found that, when collected in the near field by a high–numerical aperture objective, the scattering fringes of a flying particle with divergent diffraction angles are encoded with distinct Doppler frequency shifts—this forms a broadband “scattering rainbow” in the transverse direction relative to the incident beam axis. This angle-dependent Doppler effect transforms the spatial diffraction pattern of a flying particle into the frequency domain, enabling high-precision determination of diameter and refractive index of airborne particles within a millisecond-scale observation window. Our findings unlock degrees of freedom for resolving flying particle features in the frequency domain, establishing a versatile diagnostic platform for a broad spectrum of applications that demand in situ particle analysis, such as atmospheric aerosol monitoring, hypersonic flow diagnostics, and label-free flow cytometry.

## Teaser

Flying particles’ scattering fringes encode Doppler shifts, forming a transverse rainbow for high-precision Doppler spectrometry.

## INTRODUCTION

The scattering of micro- and nanoparticles lies at the core of light-matter interactions (*1*–*4*). Numerous emerging fields in optical physics are associated with the coherent scattering of nanostructures and materials, including metasurfaces (*5*, *6*), Mie-tronics (*7*, *8*), diffractive optics (*9*, *10*), and stimulated Raman spectroscopy (*11*), to name a few. First established by Gustav Mie in 1908, the standard Mie theory serves as a rigorous analytical solution to Maxwell’s equations, explicitly describing the scattering patterns of spherical particles across different orders (*12*). Its ability to model spherical or near-spherical particles (via approximations) renders the Mie theory indispensable in diverse disciplines such as nanotechnology (*13*, *14*), material sciences (*4*, *15*), biomedical sensing (*16*, *17*), etc. Furthermore, Mie scattering underpins modern particle metrology: The scattering patterns of particles carry intrinsic material and structural features originated from the course of light-matter interactions (*18*–*21*). In this framework, conventional scattering metrology is typically restricted to particles that are either anchored to a substrate (*22*) or levitated under constrained dynamic conditions [e.g., via optical trap potentials (*21*, *23*)]. In these well-controlled setups, data acquisition windows of several minutes can readily be allocated to integrate the inherently weak scattering signals from the test particles, ensuring the attainment of satisfactory measurement accuracy (*24*, *25*). For fast-moving particles, however—especially those dispersed in the unconfined open atmosphere—the particles’ rapid motion renders sufficient signal integration time unachievable, which, in turn, leads to substantial compromise of the accuracy for quantifying critical particle parameters (*26*, *27*). Meanwhile, direct imaging methodologies relying on camera-based detection are prone to severe motion-induced blurring in scattered-light images (*28*, *29*), with their performance ultimately constrained by the intrinsic imaging resolution of the detection system.

Here, we tackle this key challenge by first unveiling a Doppler-encoded Mie scattering effect. We show that the scattering fringes of fast-moving particles—captured at different detection angles in the near field—are imprinted with correlated Doppler-induced frequency shifts, manifesting as a broadband rainbow of purely elastic scattered light. This effect enables a transverse Doppler spectrometry (TDS), capable of quantifying the diameter and refractive index of flying particles with a millisecond observation window. At the fundamental level, the motion of the particle stimulates coherent modulation of the optical path and, consequently, the instantaneous phase of the scattered beam, introducing a frequency shift depending on the detection angle of the beam. Although angle-dependent Doppler effects have been identified for macroscopic objects (*30*–*33*), they have not been observed in the scattering patterns of micro- and nanoparticles. This gap is attributed to two key challenges: First, the very small diffraction angle of particle-scattered light collected under far-field detection, which substantially reduces the collection signal-to-noise ratio (SNR) and limits the measurable Doppler shift in the resulting spectrum because the transverse Doppler frequency shift strongly depends on the scattering angle; second, the lack of techniques to precisely control the motion and trajectory of individual flying particles. In this work, we address these limitations by leveraging state-of-the-art optical propulsion in hollow-core fibers (*34*–*38*), combined with a high–numerical aperture (NA) objective to collect dynamically scattered light in the near field from the side of the fiber. The expanded collection angle afforded by this setup substantially enhances the contribution of angle-dependent Doppler effect.

## RESULTS

**Fig. 1.**
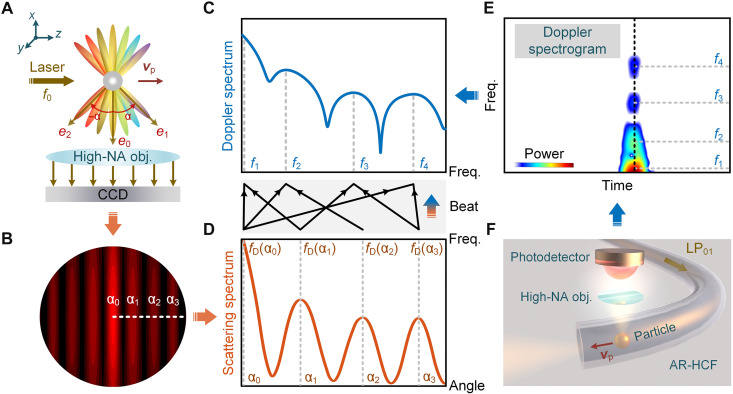
Doppler-encoded light scattering effect. (**A**) Conceptual illustration of the Doppler-encoded light scattering rainbow of flying particles. Scattered light at different angles α from a flying particle (with velocity ***v***_p_) exhibits distinct Doppler frequencies, each corresponding to a specific lobe of the particle far-field scattering pattern collected by a high-NA objective lens. (**B**) Schematic of the typical Mie scattering pattern (holographic phase function) of a dielectric microparticle, as measured by a CCD camera. (**C**) Transverse Doppler spectrum of the flying particle, derived from the beating of the measured scattering spectrum in (D). (**D**) Scattering spectrum of the flying particle. (**E**) Typical transverse Doppler spectrogram acquired by the photodetector depicted in (F). (**F**) Sketch of the experimental setup, using optically propelled particles in AR-HCF. A high-NA objective lens and photodetector are positioned laterally to the fiber to collect the transverse Doppler signal.

The Doppler-encoded Mie scattering rainbow of flying particles is observed using a dual-beam trap configuration in an antiresonant hollow-core fiber (AR-HCF). In this configuration, the particles are optically levitated by optical gradient forces in the hollow core and are propelled over the fiber through unbalancing the scattering forces of the counterpropagating beams (Fig. 1F; see Materials and Methods for the detailed setup). During the particle propulsion, a high-NA objective lens and a photodetector is placed on the side of the AR-HCF to collect the transverse scattering of the particle. The unique capability of long-distance particle propulsion in AR-HCF is highly suitable for the observation of Doppler-encoded scattering phenomenon because the particle can be propelled with a regulated speed and passing the detection area of the objective lens with well-defined trajectories (*35*, *38*, *39*).

The scattering pattern of a particle with diameter *d*_p_ comparable to laser wavelength λ appears as discrete fringes according to the standard Mie theory (Fig. 1A). The periodicity and number of fringes in the pattern are closely related to the particle diameter and its refractive index. Given the direction of incident laser beam (for instance, along the +*z* direction in Fig. 1A), the scattered light of the flying particle moving with velocity ***v***_p_ emits over divergent angles, appearing as discrete intensity patterns when collected by a camera from the side (Fig. 1B). The detected frequency of scattered beams along direction of ***e***_1_ with scattering angle α can be written as (see Supplementary Note 1 for the derivations)

$$f_{\alpha }=f_{0}\left[1+\frac{v_{p}\cdot \left(e_{1}-e_{0}\right)}{c}\right]=f_{0}+\frac{v_{p}\text{sin}\alpha }{\lambda }$$ (1)

where *f*_0_ is the incident light frequency, ***e***_0_ is the unit vector in the direction orthogonal to the beam axis in which Doppler frequency vanishes, and *c* is the vacuum speed of light. To measure the Doppler frequency shift, the scattered signal from angle −α (with direction ***e***_2_) is simultaneously captured with the same photodetector

$$f_{-\alpha }=f_{0}\left[1+\frac{v_{p}\cdot \left(e_{2}-e_{0}\right)}{c}\right]=f_{0}-\frac{v_{p}\text{sin}\alpha }{\lambda }$$ (2)

The beating frequency between *f*_α_ and *f*_−α_ gives the observed Doppler frequency shift

$$f_{D}\left(\alpha \right)=|f_{\alpha }-f_{-\alpha }|=\frac{v_{p}|e_{1}-e_{2}|}{\lambda }=\frac{2v_{p}\text{sin}\alpha }{\lambda }$$ (3)

It can be seen that *f*_D_ strongly depends on the scattering angle α, as illustrated in Fig. 1D. The collected scattering spectrum from the flying particle, after beating with each other, can further correlate to the Doppler frequency shift observed in the transverse Doppler spectrogram, as sketched in Fig. 1 (C to E). In the axial direction (+*z* direction, α = π/2), as a particular case of Eq. 3, the measured Doppler frequency shift *f*_D,z_ is proportional to the particle axial velocity *v*_p,z_

$$f_{D,z}=\frac{2}{\lambda }v_{p,z}$$ (4)

When both the incident laser beams along +*z* and −*z* are present, the measured Doppler shift collected in all directions remains as *f*_D,z_ (see Supplementary Note 1 for the derivations), which is the normal situation of far-field Doppler velocimetry reported in the literature (*40*–*42*).

### Angle-dependent transverse Doppler frequency shift

**Fig. 2.**
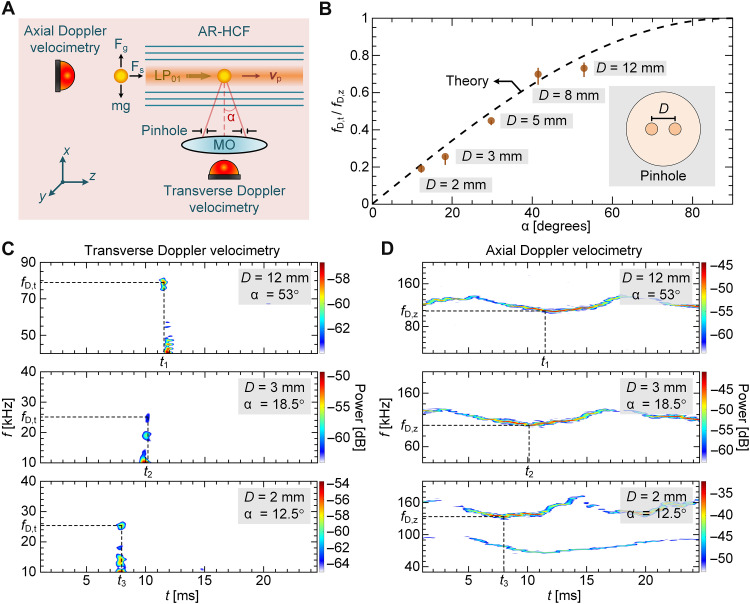
Angle-dependent transverse Doppler shift. (**A**) Sketch of the experimental configuration using AR-HCF. (**B**) Measured transverse Doppler frequency shift normalized by axial Doppler frequency *f*_D,t_/*f*_D,z_ (dots) versus the scattering angle α for various pinhole spacings (*D* = 2, 3, 5, 8, and 12 mm). Error bars are derived from the uncertainties in determining the Doppler frequency shift in both the transverse and axial Doppler spectrograms. The dashed line plots the theoretical prediction from Eq. 3. Inset: Sketch of the adopted pinholes. (**C**) Measured transverse Doppler spectrogram for the propelled 2-μm-diameter polystyrene bead in the hollow core, when the spacing *D* between the pinholes are 12 mm (top), 3 mm (middle), and 2 mm (bottom), respectively. The vertical dashed lines mark the corresponding moments of time when the particle passes the central position of the FOV of objective lens. The horizontal dashed lines mark the value of observed transverse Doppler frequency shift. (**D**) Measured axial Doppler spectrogram for the same particle as that in (C).

To observe the angle-dependent Doppler frequency effect of flying particles in the near field, an objective lens with an NA of 0.8 was placed at the side of the AR-HCF (Fig. 1F). A polystyrene bead (2 μm in diameter; *d*_p_ = 2 μm) was first trapped and propelled by the laser beam coupled into the fundamental core mode from one fiber end. The axial propulsion velocity is determined by the explicit balance between the optical scattering force and viscous drag force. The particle propulsion velocity measured via the axial Doppler velocimetry is ~5 cm/s, yielding a sampling time of ~0.3 ms for the transverse Doppler measurement—corresponding to the duration required for the particle to traverse the field of view (FOV) of the objective lens. Two pinholes (1 mm in diameter) with different spacings (*D*) were placed in front of the objective lens so that the photodetector only collects the side-scattered light from the determined angles α (see Fig. 2A). Figure 2C plots the observed Doppler spectrogram of the propelled polystyrene bead (2 μm in diameter) for the case of *D* = 12, 3, and 2 mm, respectively. The corresponding values of angle α is calculated given the working distance (4.5 mm) of the objective lens. The moments of time when the particle passes the central position of the FOV are marked as *t*_1_, *t*_2_, and *t*_3_ in the three panels. Clear Doppler frequency ingredients can be observed in the transverse Doppler spectrogram. The value of transverse Doppler shift (*f*_D,t_ in Fig. 2C) is given by the maximum frequency centered at the corresponding moment of time because other frequency components are induced by the beating of the scattered light within the pinhole due to its finite width (see Supplementary Note 2 for details).

As a comparison, the axial Doppler velocity *f*_D,z_ of the same propelled particle is also monitored by collecting the backscattered light from the fiber endface (Fig. 2D). The multiple Doppler frequencies visible in the bottom panel of Fig. 2D are induced by the simultaneous propulsion of two particles in the hollow core, whereas the transverse Doppler signals from different particles can be well separated (see Supplementary Note 5 and fig. S6 for details). The observed transverse Doppler shift (*f*_D,t_ in Fig. 2C), after normalizing with the measured axial velocity at the corresponding moment of time (i.e., *f*_D,t_/*f*_D,z_), appearing as a strong function of the scattering angle α (see dots in Fig. 2B). The dependency agrees excellently with the theoretical prediction from Eq. 3 (dashed line in Fig. 2B), validating the presence of angle-dependent Doppler effect of the flying particle. The error bars of the measured data points in Fig. 2B are determined by the uncertainties of measured Doppler frequency shift in both the transverse and axial Doppler spectrogram. Note that the effect of particle propulsion velocity variations caused by modal beating is negligible because the observation window width (given by the FOV) is more than two orders of magnitude times shorter than the fiber beat length. The precise control of the particle trajectory and velocity within the AR-HCF also ensures the reproducibility of the measured transverse Doppler spectra across repeated particle loading cycles (see Supplementary Note 4 for details). The result infers that the axial propulsion velocity of particle can also be retrieved by the transverse Doppler measurement through a proper conversion using Eq. 3, enabling three-dimensional velocity tracking of the flying particles from the side of the fiber.

### Doppler-encoded Mie scattering effect

**Fig. 3.**
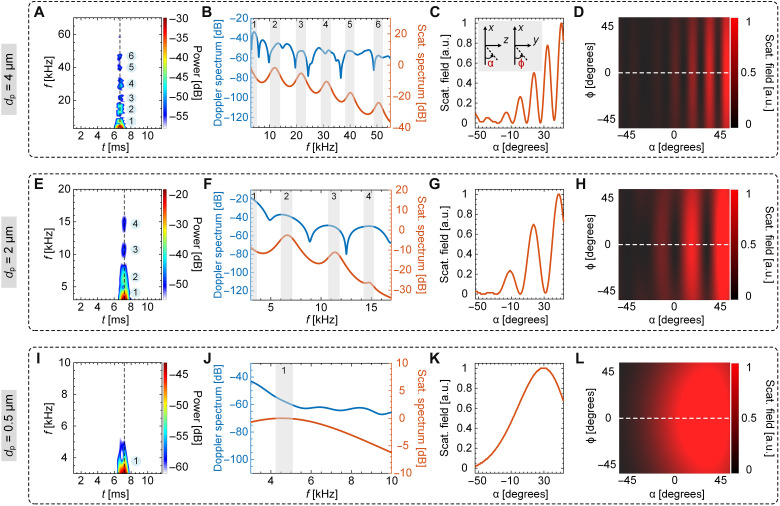
Doppler-encoded Mie scattering rainbow. (**A**) Measured Doppler spectrogram of the optically propelled 4-μm-diameter polystyrene bead in the hollow core when the particle passes the sample area of the objective lens. (**B**) Comparison of the measured Doppler spectrum (blue) along the dashed line in (A) and the converted scattering spectrum (orange) of the 4-μm-diameter polystyrene bead. The shaded areas indicate the peaks in the Doppler spectrum with the numbers correlated with that in (A). (**C**) Normalized scattering electric field of particle scattering patterns along the white-dashed line in (D). Inset: Definition of angles α and ϕ in (D); the beam propagates along the *z* axis, with the coordinate system consistent with the definition in Fig. 2A. a.u., arbitrary units. (**D**) Optical image of the calculated Mie scattering pattern of the 4-μm-diameter polystyrene bead by using the high-NA objective lens. (**E** to **H**) Results for the propelled 2-μm-diameter polystyrene bead. (**I** to **L**) Results for the propelled 500-nm-diameter polystyrene bead.

When all scattered light within the FOV of the high-NA objective lens is collected, the measured Doppler spectrogram of the propelled particle exhibits apparent discrete components in the frequency domain. This is visible in Fig. 3A in which the result of the 4-μm-diameter polystyrene bead is displayed. To understand the reason of those discrete frequencies (marked as numbers), the lateral scattering patterns collected by the same objective lens were calculated using the standard Mie theory (see Supplementary Note 3 for details). As shown in Fig. 3D, the scattered field distribution shows periodic features in the far field due to interference from the front and rear surfaces of the particle [also known as the holographic phase function (*43*)]. In this situation, the widest scattering angle is given by the collection angle of the objective lens (α = 53°). To directly correlate with the measured discrete frequency ingredients in the Doppler spectrogram (Fig. 3A), the grayscale value of scattering patterns within the FOV—along the horizontal cut line shown in Fig. 3D and proportional to the scattered electric field—is plotted in Fig. 3C. The scattering angle α can correlate to the Doppler frequency shift by using Eq. 3. As illustrated in Fig. 1 (C and D), the Doppler frequencies of all lobes of the scattering pattern can then beat with each other to generate the observed spectrum. The predicted scattering spectrum is plotted as the orange line in Fig. 3B, where the blue line represents the measured Doppler spectrum along the dashed line in Fig. 3A. It is evident that the spectral components predicted from the Mie scattering fringes are highly consistent with the observed Doppler frequency shifts (as highlighted by the shaded areas), confirming that the scattering patterns of the flying particle are encoded with the Doppler frequencies. The discrepancy in peak positions of the blue and orange lines in Fig. 3B may be attributed to perturbations of the particle scattering fringes by the cladding structure of the AR-HCF (see fig. S1A for the fiber structure), introducing multiple reflections and refractions in the beam path and hence additional dips in the observed pattern. Experiments were then repeated using a polystyrene bead with *d*_p_ = 2 μm (Fig. 3, E to H) and *d*_p_ = 500 nm (Fig. 3, I to L). It can be seen that variations in particle diameter result in distinct differences in both the number and width of frequency components in the observed Doppler spectrogram, aligning with the calculated Mie scattering fringes.

### Transverse Doppler spectrometry

**Fig. 4.**
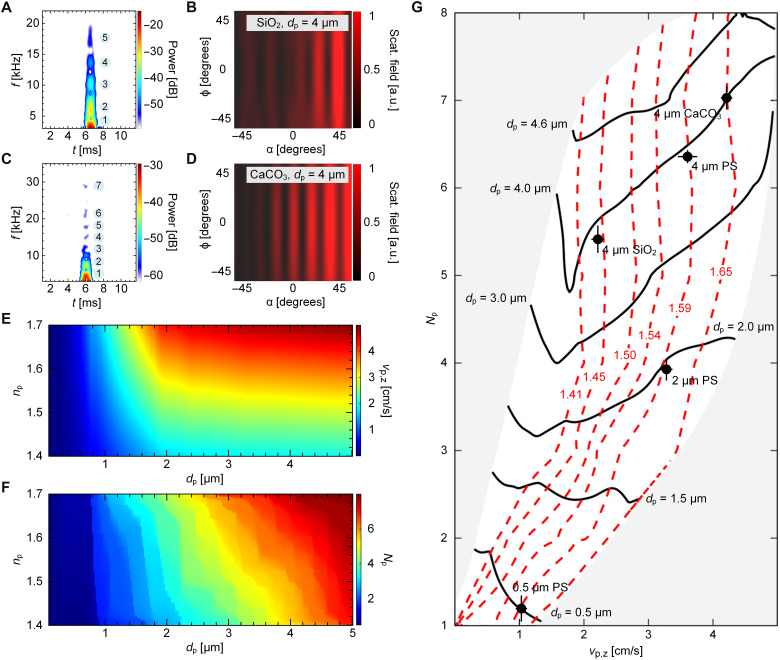
Transverse Doppler spectrometry. (**A**) Measured transverse Doppler spectrogram of the optically propelled 4-μm-diameter SiO_2_ particle and (**B**) its calculated lateral scattering pattern using the Mie theory. (**C** and **D**) Results for the optically propelled 4-μm-diameter CaCO_3_ particle. (**E**) Calculated axial particle velocity *v*_p,z_ and (**F**) the number of fringes *N*_p_ as a function of particle diameter *d*_p_ and refractive index *n*_p_. (**G**) Contour plots of particle diameter *d*_p_ (solid black) and refractive index *n*_p_ (dashed red) as a function of particle axial velocities *v*_p,z_ and the number of scattering fringes *N*_p_ resolved by the transverse Doppler spectrogram. The gray-shaded area denotes the region where no physically valid solutions exist for particle diameter and refractive index. The black dots correspond to the measured *v*_p,z_ and *N*_p_ values for particles with specified *d*_p_ and *n*_p_, with error bars representing the associated measurement uncertainties.

The resolved Doppler-encoded light scattering effect, acted as a fast Doppler camera to capture particle diffraction patterns, unlocks possibilities for characterizing flying particles. Figure 4 (A and C) plots the measured transverse Doppler spectrogram of 4-μm-diameter SiO_2_ and CaCO_3_ particles, respectively. It can be observed that the number of frequency components counted in the Doppler spectrogram is 5 for SiO_2_ particles and 7 for CaCO_3_ particles—consistent with the number of lobes in the simulated scattered electric fields (Fig. 4, B and D). Because both the axial propulsion velocity and the scattering patterns resolved by transverse Doppler spectrogram are strong function of particle diameter *d*_p_ and refractive index *n*_p_ (Fig. 4A), the technique may be exploited to the simultaneously retrieve *d*_p_ and *n*_p_ of flying particles. To demonstrate this capability, the axial propulsion velocity (*v*_p,z_) and the number of scattering patterns fringes (*N*_p_) were calculated as functions of *d*_p_ and *n*_p_. Specifically, *v*_p,z_ was determined by explicitly balancing the optical scattering force against the viscous drag force exerted on the particle. The results in Fig. 4E indicate that *v*_p,z_ increases with *d*_p_ and *n*_p_, primarily because the optical scattering force depends more strongly on *d*_p_ and *n*_p_ than the viscous drag force. The dependence of *N*_p_ on *d*_p_ and *n*_p_ was calculated using the standard Mie theory (see Supplementary Note 3 for details), with the results presented in Fig. 4F. Because of the limited FOV of the objective lens, the measured scattering fringes may appear incomplete at the FOV boundary, as shown in Fig. 4D. In such cases, a width extraction algorithm was used to calculate the percentage of the partial fringe width at the boundary relative to the average fringe width across the entire pattern (see Materials and Methods for details). This enables a continuous variation of *N*_p_, improving the accuracy of *d*_p_ and *n*_p_ retrieval.

On the basis of the results in Fig. 4 (E and F), Fig. 4G presents the calculated contour lines for *d*_p_ (solid black) and *n*_p_ (dashed red), plotted as functions of *v*_p,z_ and *N*_p_. The intersections of the black and red contour lines correspond to the retrieved *d*_p_ and *n*_p_ for the given *v*_p,z_ and *N*_p_. Notably, the contour lines for constant *d*_p_ and constant *n*_p_ are nearly orthogonal, indicating that the proposed TDS can unambitiously resolve flying particle diameter and refractive index with high precision. The capability to resolve particle refractive index can be leveraged to infer the material properties of particles in situ. In the contour lines of constant *d*_p_, the increase in *N*_p_ at smaller *v*_p,z_ is attributed to the splitting of scattering fringes into dual peaks originated from the solution of the Mie theory. The gray-shaded area denotes the forbidden region, where no valid *d*_p_ and *n*_p_ solutions exist for a given combination of *v*_p,z_ and *N*_p_. Thus, particles with higher propulsion velocities are associated with larger *d*_p_ and *n*_p_ and, consequently, an increasing *N*_p_. The dots in Fig. 4G represent the measured *v*_p,z_ and *N*_p_ for particles with known *d*_p_ and *n*_p_, showing reasonable agreement with the calculated contour lines. Horizontal error bars denote the uncertainties associated with the determination of the axial Doppler frequency shift (and thus of *v*_p,z_), whereas vertical error bars stem from the uncertainties in measuring the average fringe width of the scattering pattern.

## DISCUSSION

**Fig. 5.**
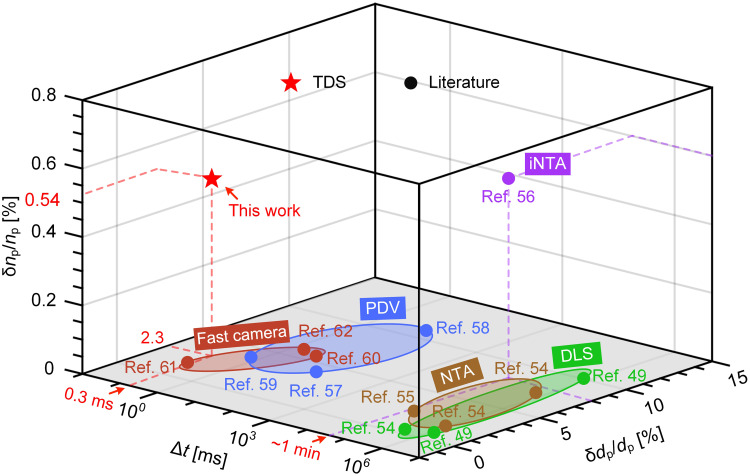
Comparison of TDS and prior techniques for flying particle characterization. The TDS technique is compared with common methods (DLS, NTA, PDV, and fast camera) for characterizing freely moving particles, based on the relevant literature (*49*, *50*, *54*–*63*). All methods without refractive index retrieval capability are confined to the Δ*t*-δ*d*_p_/*d*_p_ plane, as indicated by the gray-shaded surface.

Flying particle metrology is in high demand across diverse scenarios, including atmospheric aerosol characterization (*36*, *44*), hypersonic aerodynamic research (*28*, *45*), and the development of inhalable nanomedicines (*46*, *47*). The TDS approach presented in this work, by leveraging optical forces within AR-HCF for precise manipulation of single particles, offers a versatile solution for characterizing flying particles even when data acquisition time is severely limited. Figure 5 presents a comparison of the proposed TDS technique against several established methods for flying particle characterization, based on three key metrics: data acquisition time (Δ*t*), relative accuracy of diameter (δ*d*_p_/*d*_p_), and refractive index measurement (δ*n*_p_/*n*_p_). All methods without refractive index retrieval capability are confined to the Δ*t*-δ*d*_p_/*d*_p_ plane, as indicated by the gray-shaded surface. Specifically, dynamic light scattering (DLS) has been developed to analyze the size distribution of nanoparticles suspended in solution. However, DLS measures ensembles of diffusing particles and has limited capability of single-particle characterization (*48*–*50*). Although the quasielastic light scattering technique has been proposed to realized single-particle characterization, its temporal resolution is limited to the minute level because it requires analyzing the temporal fluctuations of scattered light induced by Brownian motion to construct an autocorrelation function (*51*, *52*). This makes it inadequate for the rapid and continuous characterization of flying particles. Nanoparticle tracking analysis (NTA), by contrast, can track the trajectories of individual particles, and by quantifying the particle’s diffusion constant, its hydrodynamic diameter can be retrieved (*53*–*56*). Recent advancements have combined NTA with interferometric scattering approaches (iNTA), enabling simultaneous measurements of the size and refractive index of diffusing particles (*57*). Notably, both DLS and NTA techniques are restricted to aqueous environment, where the Brownian motion of diffusing particles is confined to a limited range due to solution viscosity. For this operating scenario, both techniques demand a minute-level acquisition window to enable video recording and statistical evaluation of repeated measurements, a prerequisite for securing satisfactory diameter measurement accuracy. In the open atmosphere, the transient nature of flying airborne particles—resulting from their much faster motion—severely constrains available metrology tools. Phase Doppler velocimetry (PDV) has been established by detecting the phase difference of Doppler signals in the airborne particle scattered light from different angles to probe the speed and diameter distribution of the sprayed aerosols (*58*, *59*) and plasma particles (*60*). Nevertheless, PDV exhibits limited measurement accuracy for smaller particles due to their substantially weaker scattered intensity, and the size determination requires prior knowledge of the particle refractive index. Most recently, fast cameras (with frame rates ranging from kilohertz to megahertz) have been used to track the trajectories of the flying objects (*61*–*63*). The optimal diameter measurement accuracy of the fast camera is given by the resolution limit of the imaging system (some hundreds of nanometers). As illustrated in Fig. 5, the TDS’s ability to simultaneously determine both the diameter and refractive index of flying particles—coupled with its millisecond-scale observation window—is expected to substantially enhance the characterization throughput and accuracy of flying particle analysis. Although validated for airborne particles, the technique can be reliably extended to aqueous-phase particle analysis, facilitating applications such as high-throughput, label-free flow cytometry for single-cell analysis (*64*).

As a type of transverse Doppler velocimetry, the technique also holds promise for probing in-plane motion—and consequently the three-dimensional motion—of moving objects, thereby enhancing the capabilities of laser Doppler measurement. The resolution of particle velocity measurement can be improved by further increasing the accuracy of Doppler frequency tracking and enhancing the SNR of the transverse Doppler signal. In the context of levitated optomechanics (*65*), the technique provides an approach for resolving the three-dimensional Brownian motion of levitated particles and for realizing novel protocols for macroscopic particle cooling in an optical trap.

## MATERIALS AND METHODS

### Experimental setup

The schematic of the experimental setup is presented in fig. S1A. A 1064-nm continuous-wave laser (Laser 1) served as the optical trapping light source. A half-wave plate (HWP) and a polarizing beam splitter (PBS) were used to adjust the power balance between two separated trapping beams with orthogonal state of polarizations (s- and p-polarization). Both beams were then coupled into the fundamental mode (LP_01_ mode) of an AR-HCF via a coupling lens. A green laser (Laser 2), introduced into the setup through a dichroic mirror (DM), was used as an illumination source for tracking the particle trajectory in the fiber and for aligning the transverse detection system. In the measurement, a charge-coupled device (CCD) camera was positioned at the side of the fiber to monitor the particle loading and propulsion process. Because the camera exhibits a higher response at a 532-nm wavelength than at 1064 nm, Laser 2 was used to monitor the particle position during its propulsion (see fig. S1, B and C, in the Supplementary Materials). This facilitated precise alignment of the transverse Doppler detection system with the flying particle, ensuring an optimal SNR for the transverse Doppler signals acquired with the 1064-nm laser. Two photodetectors (PD1 and PD2) were positioned near the endface and laterally to the AR-HCF, respectively, for collecting backscattered and side-scattered light from propelled particles containing the axial and transverse Doppler signals. In the measurement, a meshed nebulizer was used to deliver an aqueous dispersion of polystyrene beads (in distilled water) near the fiber endface. The reflection signal detected by PD1 was used to verify whether particles have interacted with the trapping beam. Once the conditions for the stable particle trapping were satisfied (*35*, *66*), a droplet containing the particle can be levitated close to the fiber endface; subsequent water evaporation left a solid particle ready for manipulation. The inset of fig. S1A shows the cross section of the AR-HCF used in the experiment, overlaid with the measured intensity distribution of the excited fundamental core mode. The core and cladding diameters of the AR-HCF are 28 and 285 μm, respectively.

Figure S1B shows an optical image of a levitated 2-μm-diameter polystyrene bead near the fiber endface. By rotating the HWP to adjust the power ratio of the counterpropagating trapping beams, the particle could be robustly loaded into the fiber core (fig. S1C). Particle propulsion was achieved by simply blocking one of the trapping beams. A high-NA (NA = 0.8) objective lens was placed laterally to the fiber, in front of PD2, for collecting near-field side-scattered light. Two pinholes (1 mm in diameter) with different spacings *D* were positioned in front of the high-NA objective lens, ensuring that PD2 only collects the side-scattered light from the determined angles α. As the particle traversed the objective’s FOV, the voltage signal collected by PD2 increased. Subsequently, a short-time Fourier transform was applied to the time-domain voltage signal to retrieve the Doppler spectrogram of the flying particle.

### Extraction of the partial scattering fringe width at the FOV boundary

To enable a continuous variation of number of fringes *N*_p_ for the test particles, a procedure is established to extract the width of incomplete scattering fringes at the FOV boundary. As an example, the normalized scattering field extracted from the calculated Mie scattering pattern for a spherical particle with *d*_p_ = 4.1 μm and *n*_p_ = 1.60 is plotted in fig. S4. All local maxima (orange dots) and minima (green dots) of the curve were first identified. The average fringe width *W* was then estimated by taking the mean width of all complete fringes. For partial fringes at the boundary, their effective width was estimated on the basis of the fractional length of the incomplete portion (gray-shaded areas in fig. S4) relative to *W*, yielding an effective count for these partial fringes

$$N_{b}=\frac{\alpha_{1}-\alpha_{L}}{W}+\frac{\alpha_{R}-\alpha_{n}}{W}$$ (5)

where α_1_ and α*_n_* denote the collection angle of the 1st and *n*th complete fringes, and α_L_ and α_R_ represent left and right boundaries of the FOV, respectively. The effective fringe number *N*_p_ for the particle was then calculated as the sum of *N*_b_ and total number of complete fringes across the entire scattering pattern.

## Supplementary Materials

### This PDF file includes:

Supplementary Notes S1 to S5 Figs. S1 to S6 References
