# Supplementary material for: Doppler-encoded Mie scattering rainbow of flying particles
Source: Sci Adv. 2026 Jun 17;12(25):eaef7659. doi: 10.1126/sciadv.aef7659 (PMC13274609; doi:10.1126/sciadv.aef7659)
Supplement: Supplementary file 1 — Supplementary Notes S1 to S5 Figs. S1 to S6 References [file sciadv.aef7659_sm.pdf]

Science Advances
AAAS

Supplementary Materials for

Doppler-encoded Mie scattering rainbow of flying particles

Rui Wang *et al.*

Corresponding author: Shangran Xie, sxie@bit.edu.cn

Sci. Adv. **12**, eaef7659 (2026)
DOI: 10.1126/sciadv.aef7659

This PDF file includes:

Supplementary Notes S1 to S5
Figs. S1 to S6
References

Supplementary Note 1: Derivation of angle-dependent Doppler frequency shift induced by a flying particle

The Doppler effect manifests in light scattered by a moving particle. When a laser beam is incident on a moving particle, as illustrated in Fig. S2A, the observed frequency (f_1) of the scattered beam in direction $\boldsymbol{e}_0$ is derivable from the relativistic theory (*67*):

$$f_1 = f_0 \frac{1 - \dfrac{\boldsymbol{v}_\mathrm{p} \cdot \boldsymbol{e}_0}{c}}{\sqrt{1 - (\dfrac{\boldsymbol{v}_\mathrm{p} \cdot \boldsymbol{e}_0}{c})^2}} \tag{S1}$$

where f_0 is incident light frequency, $\boldsymbol{e}_0$ is unit vector in the direction orthogonal to the beam axis in which Doppler frequency vanishes, c is the vacuum speed of light, $\boldsymbol{v}_\mathrm{p}$ is the velocity of moving particles. Since c>> $\boldsymbol{v}_\mathrm{p}\cdot\boldsymbol{e}_0$, Eq. (S1) can be simplified as:

$$f_1 = f_0 \left(1 - \frac{\boldsymbol{v}_\mathrm{p} \cdot \boldsymbol{e}_0}{c} \right) \tag{S2}$$

When a photodetector collects the scattered light of flying particle from a fixed position in direction $\boldsymbol{e}_1$, the observed frequency f_2 can be written as:

$$f_2 = f_1 \left(1 + \frac{\boldsymbol{v}_\mathrm{p} \cdot \boldsymbol{e}_1}{c} \right) \tag{S3}$$

substitute Eq. (S2) into Eq. (S3):

$$f_2 = f_0 \left(1 + \frac{\boldsymbol{v}_\mathrm{p}}{c} \cdot (\boldsymbol{e}_1 - \boldsymbol{e}_0) - \frac{\boldsymbol{v}_\mathrm{p}^2 \cdot \boldsymbol{e}_1 \cdot \boldsymbol{e}_0}{c^2} \right) \tag{S4}$$

since c^2>> $\boldsymbol{v}_\mathrm{p}^2\cdot\boldsymbol{e}_1\cdot\boldsymbol{e}_0$, the expression of f_2 can be simplified as:

$$f_2 = f_0 \left(1 + \frac{\boldsymbol{v}_\mathrm{p}}{c} \cdot (\boldsymbol{e}_1 - \boldsymbol{e}_0) \right) \tag{S5}$$

It is then possible to derive the observed Doppler frequency shifts of the scattered beams with different scattering angles with respect to the incident beams. Eq. (1) and Eq. (2) of the main text are derived from Eq. (S5).

When two beams are simultaneously incident onto the moving particle (with included angle 2β), as sketched in Fig. S2B, the frequencies of scattered light in $\boldsymbol{e}_1$ and $\boldsymbol{e}_2$ directions are:

$$f_1 = f_0 [1 + \frac{\boldsymbol{v}_\mathrm{p} \cdot (\boldsymbol{e}_1 - \boldsymbol{e}_0)}{c}] \tag{S6}$$

$$f_2 = f_0 [1 + \frac{\boldsymbol{v}_\mathrm{p} \cdot (\boldsymbol{e}_2 - \boldsymbol{e}_0)}{c}] \tag{S7}$$

then the Doppler frequency shift is beat frequency of two scattered beams:

$$f_{\mathrm{D}} = |f_1 - f_2| = \frac{v_{\mathrm{p}}|\boldsymbol{e}_1 - \boldsymbol{e}_2|}{\lambda} = \frac{2v_{\mathrm{p}}\sin\beta}{\lambda} \tag{S8}$$

From Eq. (S8), it can be seen that the measured Doppler frequency shifts from the detector only relates to incident (rather than scattered) angles, PD can therefore be placed at any location suitable for collecting the scattered beams. When both the incident laser beams along +*z* and -*z* are present (i.e. $\beta = \pi/2$), the measured Doppler shift collected in all directions remains as $f_{\mathrm{D,z}} = 2v_{\mathrm{p,z}}/\lambda$ according to Eq. (S8).

Supplementary Note 2: Multiple frequency components in the transverse Doppler spectrogram

In the experimental setup shown in Fig. 2A of the main text, two pinholes (1 mm diameter) with different spacings (*D*) were placed in front of the high-NA objective lens so that the photodetector only collects the side-scattered light from the determined angles α. In this case, as illustrated in Fig. S3, parts of the scattering fringes of the flying particle (marked by the red lobes 1-4) can pass through the pinholes and are then heterodyned on the PD. According to Eq. (1) in the main text, each lobe has different Doppler frequency shifts correlated with the scattering angle α. The beat frequency between the ith and j^{th} lobe, thus $\Delta f_{\mathrm{i,j}} = f_{\mathrm{i}} - f_{\mathrm{j}} = v_{\mathrm{p}}(\sin\alpha_{\mathrm{i}} - \sin\alpha_{\mathrm{j}})/\lambda$. Notably, the scattering fringes collected by the same pinhole differ only slightly in the scattering angle, producing low-frequency beat notes near zero (thus, $\Delta f_{4,3}=\Delta f_{2,1}\sim 0$). In contrast, fringes from different pinholes exhibit larger frequency spacing and hence generate high-frequency components ($\Delta f_{4,2}$, $\Delta f_{3,2}$, and $\Delta f_{4,1}$) in the measured transverse Doppler spectrogram, with $\Delta f_{4,1}$ being the highest frequency component determining $f_{\mathrm{D,t}}$ in the main text. This is the reason for the observed multiple frequency components in the transverse Doppler spectrogram (Fig. 2C of the main text). The spectral width of each frequency component is intrinsically determined by the scattering pattern of the particle. When this pattern is partially blocked by the pinhole, the spectral width becomes governed by the pinhole width, as the pinhole defines the acceptance range of the scattering fringes (see Fig. S3).

Supplementary Note 3: Calculation of the particle scattering patterns using Mie theory

According to standard Mie theory, when an *x*-polarized plane wave traveling along the *z*-axis is scattered by the flying particle (see Fig. 1A in the main text for coordinate system definitions), the scattered electric field $\mathbf{E}_{\mathrm{s}}$ outside the particle can be expressed as:

$$\mathbf{E}_{\mathrm{s}} = \sum_{m=1}^{\infty} E_m \left(i a_m \mathbf{N}^{(3)}_{(\mathrm{e})lm} - b_m \mathbf{M}^{(3)}_{(\mathrm{o})lm} \right) \tag{S9}$$

where $E_m = i^m E_0(2m+1)/[(m(m+1))]$, E_0 is the amplitude of the incident electric field, *m* and *l* are positive integers. When $m \geq l$, a_m and b_m are scattering coefficients defined as:

$$a_m = \frac{w\psi_m(w\gamma)\psi'_m(\gamma) - \psi_m(\gamma)\psi'_m(w\gamma)}{w\psi_m(w\gamma)\xi'_m(\gamma) - \xi_m(\gamma)\psi'_m(w\gamma)} \tag{S10}$$

$$b_m = \frac{\psi_m(w\gamma)\psi'_m(\gamma) - w\psi_m(\gamma)\psi'_m(w\gamma)}{\psi_m(w\gamma)\xi'_m(\gamma) - w\xi_m(\gamma)\psi'_m(w\gamma)} \tag{S11}$$

where $w = n_p / n_u$ is the relative particle refractive index with respect to the surrounding medium (n_u), $\gamma = kd_p/2$ is the size parameter of the particle, k is the wave number in air. $\psi_m(z) = zj_m(z)$ and $\xi_m(z) = zh_m^{(1)}(z)$ are the Riccati-Bessel functions, j_m is spherical Bessel function of the first kind, and $h_m^{(1)}$ is spherical Hankel function of the first kind. When $l = 1$, $\mathbf{M}_{(o)lm}$ and $\mathbf{N}_{(e)lm}$ can be expressed as:

$$\begin{aligned}\mathbf{N}_{(e)1m} &= \cos\varphi m(m+1)\sin\theta\pi_m(\cos\theta)\frac{z_m(kr)}{kr}\hat{\mathbf{e}}_r + \cos\varphi\tau_m(\cos\theta)\frac{[krz_m(kr)]'}{kr}\hat{\mathbf{e}}_\theta \\ &\quad - \sin\varphi\pi_m(\cos\theta)\frac{[krz_m(kr)]'}{kr}\hat{\mathbf{e}}_\phi\end{aligned} \quad \text{(S12)}$$

$$\mathbf{M}_{(o)1m} = \cos\varphi\pi_m(\cos\theta)z_m(kr)\hat{\mathbf{e}}_\theta - \sin\varphi\tau_m(\cos\theta)z_m(kr)\hat{\mathbf{e}}_\phi \quad \text{(S13)}$$

where π_m and τ_m are angle-dependent functions. Superscripts appended to functions $\mathbf{M}_{(o)lm}$ and $\mathbf{N}_{(e)lm}$ denotes the type of spherical Bessel function, and the superscript (3) denotes spherical Hankel function of the first kind. $\hat{e}_r$, $\hat{e}_\theta$ and $\hat{e}_\varphi$ are unit vectors along the direction of r, θ and φ respectively. Using Eq. (S9-S13), the electric field distributions of the Mie scattering fringes can be calculated for particles of varying diameters and refractive indices, as shown in Fig. 3(D,H,L) and Fig. 4(B,D) of the main text.

To understand the azimuthal variation of the scattering amplitude (as observed in Fig. 3(D,H,L) of the main text), the amplitude scattering matrix elements, $S_1(\theta)$ and $S_2(\theta)$, can be calculated:

$$\begin{cases} S_1(\theta) = \sum_{m=1}^{\infty}\frac{2m+1}{m(m+1)}(a_m\pi_m + b_m\tau_m) \\ S_2(\theta) = \sum_{m=1}^{\infty}\frac{2m+1}{m(m+1)}(a_m\tau_m + b_m\pi_m) \end{cases} \quad \text{(S14)}$$

$S_1(\theta)$ and $S_2(\theta)$ represent the perpendicular (s-polarized) and parallel (p-polarized) components relative to the scattering plane, respectively. For an x-polarized incident plane wave propagating along the z-axis, the spatial distribution of the scattered intensity $I(\theta, \varphi)$ depends explicitly on the azimuthal angle φ:

$$I(\theta,\varphi) \propto |S_1(\theta)|^2\sin^2\varphi + |S_2(\theta)|^2\cos^2\varphi \quad \text{(S15)}$$

Therefore, even though the particle itself is perfectly symmetric around the z-axis, the linear polarization of the incident light breaks the azimuthal symmetry of the scattered field, causing scattering amplitude to vary with φ.

Supplementary Note 4: Reproducibility of the transverse Doppler measurements

To evaluate the reproducibility of transverse Doppler velocimetry, we performed five independent measurement runs for 2-µm-diameter polystyrene beads with a fixed pinhole spacing of $D = 3$ mm. The ratios of the transverse Doppler shift to the axial Doppler shift ($f_{D,t}/f_{D,z}$), which normalize out the influence of velocity fluctuations induced by changes in fiber coupling condition, are plotted in Fig. S5. The standard deviation of the five data points is ~13.4%, validating the reproducibility of the transverse Doppler measurements.

Supplementary Note 5: Identification of multiple particle propulsion

In the bottom panel of Fig. 2D in the main text, multiple Doppler frequencies are visible, induced by the simultaneous propulsion of two particles in the hollow core. To ensure single-particle transverse Doppler metrology, we rely on the spatial separation of the transverse Doppler signals when different particles are propelled along the fiber. Fig. S6 displays the time-domain signals collected by PD1 (red) and PD2 (blue), which represent the axial and transverse Doppler signals, respectively. The figure shows that when more than one particle is present, they traverse the detection field-of-view (FOV) of the transverse Doppler detection system at different moments, producing temporally isolated signals. By processing the Doppler signal within the corresponding time windows, the Doppler spectrum of each individual particle can be accurately extracted. Furthermore, the optical images and trajectories of the individual particles can be identified with the CCD camera positioned at the side of the fiber, which also verifies that only one particle is being analyzed at a time.

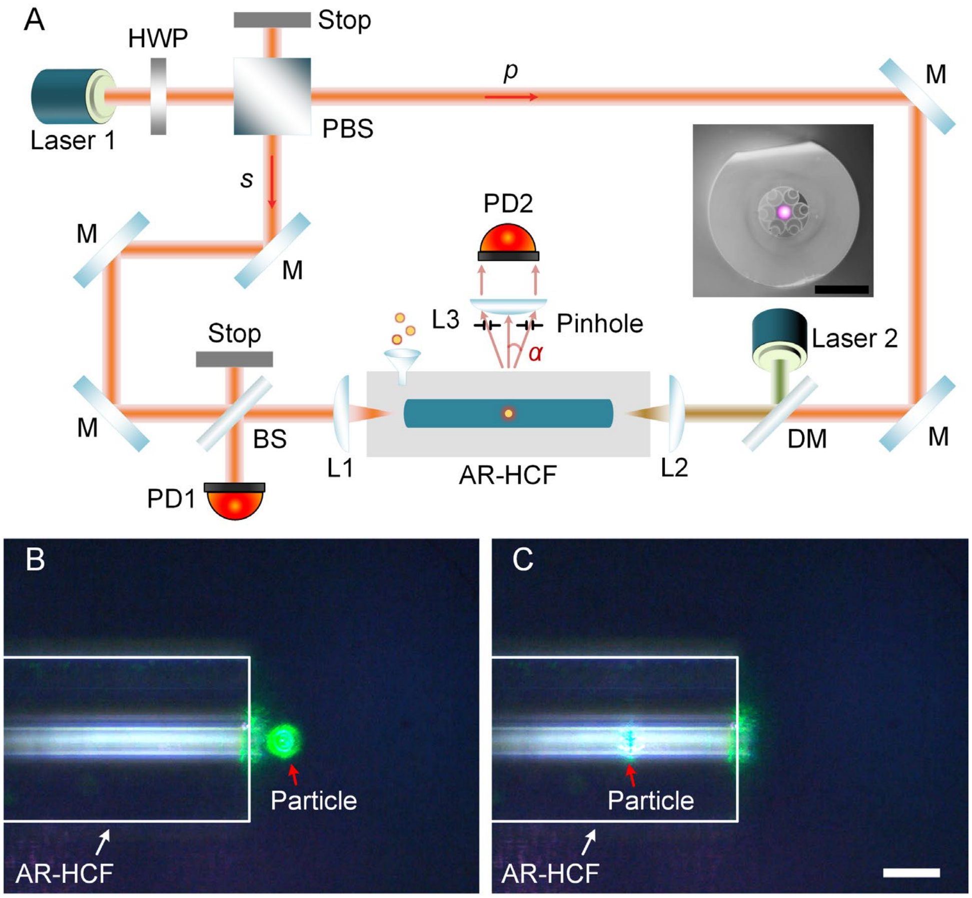

Fig. S1. Experimental setup. A, Schematic diagram of the experimental setup. HWP, half-wave plate; PBS, polarizing beam splitter; BS, beam splitter; PD, photodetector; L, lens; M, Mirror. Inset: optical image of the cross- section of the used AR-HCF overlaid with the measured intensity profile of the excited fundamental core mode. Scar bar: 100 µm. **B,** Optical image of a 2-µm-diameter polystyrene bead trapped outside and **C** inside the core of AR-HCF. Scale bar: 100 µm.

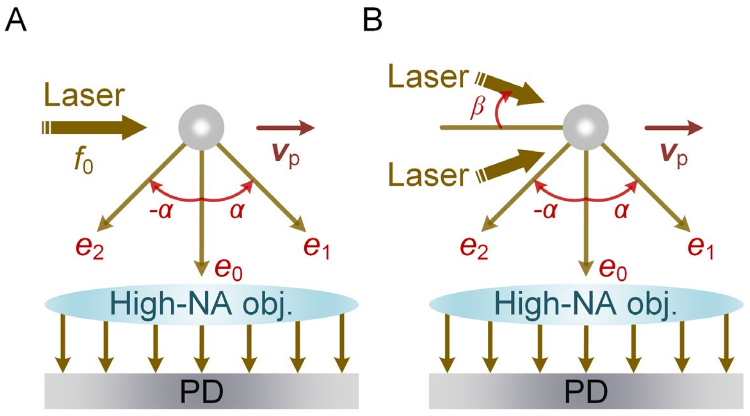

Fig. S2. Doppler-encoded light scattering effect. A, Sketch of single-incident-beam and **B,** dual-incident-beam configuration.

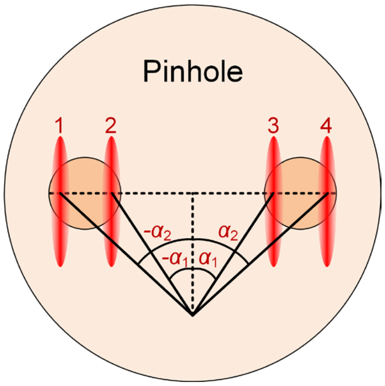

Fig. S3. Beating among different scattering fringes passing through the pinholes. The red lobes labeled 1-4 correspond to the scattering fringes collected at the respective scattering angles α of the moving particle.

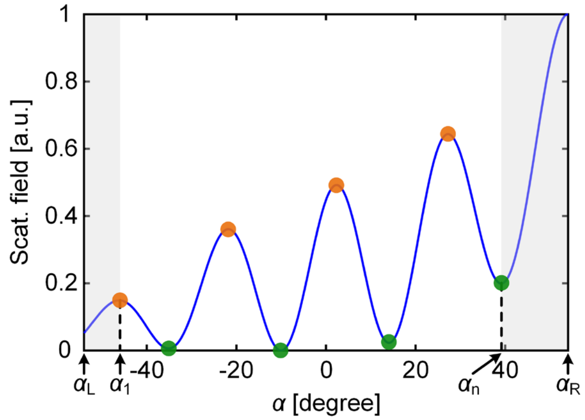

Fig. S4. Algorithm for determining the width of partial scattering fringes. The gray-shaded areas represent the incomplete portions of fringes truncated at the boundaries of the FOV.

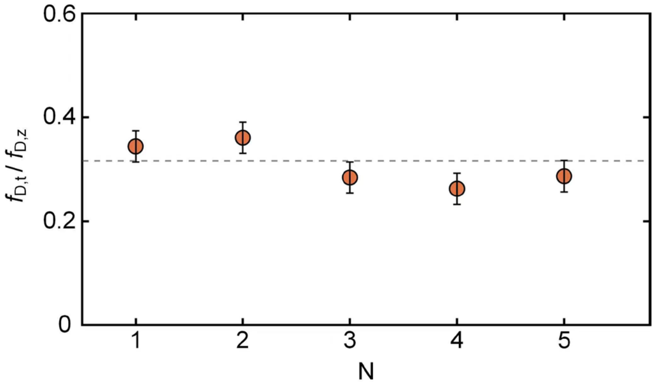

Fig. S5. Reproducibility of the transverse Doppler measurements. Measured ratio of transverse to axial Doppler frequency shifts ($f_{D,t}/f_{D,z}$) for five independent experimental runs (D = 3 mm). The dashed horizontal line indicates the value of theoretical prediction using Eq. (3). Error bars are derived from the uncertainties in determining the Doppler frequency shift in both the transverse and axial Doppler spectrograms for each individual measurement.

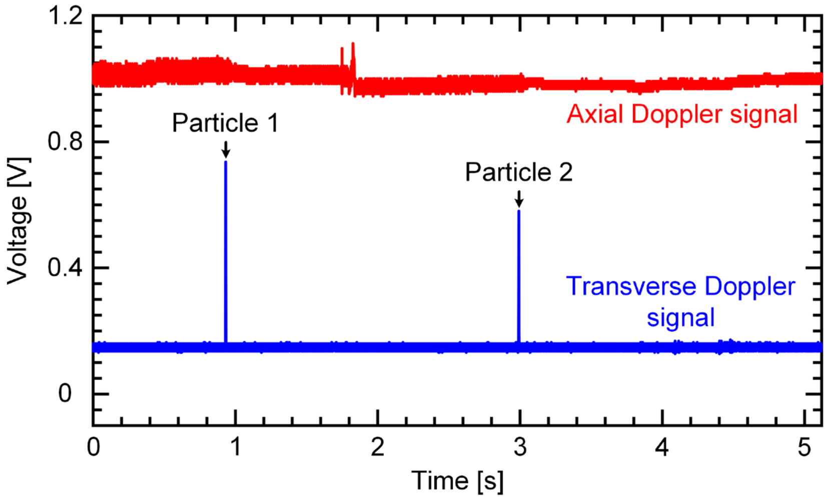

Fig. S6. Measured time-domain axial (red) and transverse (blue) Doppler signals when two particles are simultaneously propelled in the hollow core. Distinct, temporally separated transverse Doppler signals (peaks) are visible as particle 1 and 2 traverse the detection field-of-view at different moments.

REFERENCES

1. R. Hillenbrand, T. Taubner, F. Keilmann, Phonon-enhanced light–matter interaction at the nanometre scale. *Nature* **418**, 159–162 (2002).

2. N. Rivera, I. Kaminer, Light-matter interactions with photonic quasiparticles. *Nat. Rev. Phys.* **2**, 538–561 (2020).

3. A. V. Romanov, M. A. Yurkin, Single-particle characterization by elastic light scattering. *Laser Photonics Rev.* **15**, 2000368 (2021).

4. A. Dorodnyy, J. Smajic, J. Leuthold, Mie scattering for photonic devices. *Laser Photonics Rev.* **17**, 2300055 (2023).

5. H. Yu, Y. Peng, Y. Yang, Z.-Y. Li, Plasmon-enhanced light-matter interactions and applications. *npj Comput. Mater.* **5**, 45 (2019).

6. Z.-Y. Li, Mesoscopic and microscopic strategies for engineering plasmon-enhanced raman scattering. *Adv. Opt. Mater.* **6**, 1701097 (2018).

7. V. E. Babicheva, A. B. Evlyukhin, Mie-resonant metaphotonics. *Adv. Opt. Photonics* **16**, 539–658 (2024).

8. H. Barati Sedeh, N. M. Litchinitser, From non-scattering to super-scattering with Mie-tronics. *Photonics Res.* **12**, 608–624 (2024).

9. Q. Zhang, Z. He, Z. Xie, Q. Tan, Y. Sheng, G. Jin, L. Cao, X. Yuan, Diffractive optical elements 75 years on: From micro-optics to metasurfaces. *Photonics Insights* **2**, R09 (2023).

10. Y. U. Lee, S. Li, G. B. M. Wisna, J. Zhao, Y. Zeng, A. R. Tao, Z. Liu, Hyperbolic material enhanced scattering nanoscopy for label-free super-resolution imaging. *Nat. Commun.* **13**, 6631 (2022).

11. L. Sirleto, A. Vergara, M. A. Ferrara, Advances in stimulated Raman scattering in nanostructures. *Adv. Opt. Photonics* **9**, 169–217 (2017).

12. G. Mie, Beiträge zur Optik trüber Medien, speziell kolloidaler Metallösungen. *Ann. Phys.* **330**, 377–445 (1908).

13. P. J. Wyatt, Measurement of special nanoparticle structures by light scattering. *Anal. Chem.* **86**, 7171–7183 (2014).

14. Y. Kivshar, The rise of Mie-tronics. *Nano Lett.* **22**, 3513–3515 (2022).

15. I. Niskanen, V. Forsberg, D. Zakrisson, S. Reza, M. Hummelgård, B. Andres, I. Fedorov, T. Suopajärvi, H. Liimatainen, G. Thungström, Determination of nanoparticle size using Rayleigh approximation and Mie theory. *Chem. Eng. Sci.* **201**, 222–229 (2019).

16. H. Kim, H. Yun, S. Jeong, S. Lee, E. Cho, J. Rho, Optical metasurfaces for biomedical imaging and sensing. *ACS Nano* **19**, 3085–3114 (2025).

17. A. V. Kabashin, V. G. Kravets, A. N. Grigorenko, Label-free optical biosensing: Going beyond the limits. *Chem. Soc. Rev.* **52**, 6554–6585 (2023).

18. E. S. Yastrebova, I. Dolgikh, K. V. Gilev, I. V. Vakhrusheva, E. Liz, A. L. Litvinenko, V. M. Nekrasov, D. I. Strokotov, A. A. Karpenko, V. P. Maltsev, Spectral approach to recognize spherical particles among non-spherical ones by angle-resolved light scattering. *Opt. Laser Technol.* **135**, 106700 (2021).

19. U. Ortiz-Orruño, R. Quidant, N. F. van Hulst, M. Liebel, J. Ortega Arroyo, Simultaneous sizing and refractive index analysis of heterogeneous nanoparticle suspensions. *ACS Nano* **17**, 221–229 (2022).

20. J. Li, W. Zhang, A. Engarnevis, Holographic imaging platform for particle discrimination based on simultaneous mass density and refractive index measurements. *Opt. Express* **31**, 38989–39006 (2023).

21. B. Schellenberg, M. M. Behbahani, N. Balasubramanian, T. H. Fikkers, S. Hoekstra, Mass and shape determination of optically levitated nanoparticles. *Appl. Phys. Lett.* **123**, 114102 (2023).

22. R. Ceolato, K. Aleau, L. Paulien, E. Reynoso-Lara, M. J. Berg, Multispectral small-angle light scattering from particles. *Opt. Lett.* **46**, 3155–3158 (2021).

23. Y. Jin, J. Yan, S. J. Rahman, X. Yu, J. Zhang, Imaging the dipole scattering of an optically levitated dielectric nanoparticle. *Appl. Phys. Lett.* **119**, 021106 (2021).

24. D. D. Postnov, J. Tang, S. E. Erdener, K. Kılıç, D. A. Boas, Dynamic light scattering imaging. *Sci. Adv.* **6**, eabc4628 (2020).

25. B. Špačková, H. Klein Moberg, J. Fritzsche, J. Tenghamn, G. Sjösten, H. Šípová-Jungová, D. Albinsson, Q. Lubart, D. van Leeuwen, F. Westerlund, D. Midtvedt, E. K. Esbjörner, M. Käll, G. Volpe, C. Langhammer, Label-free nanofluidic scattering microscopy of size and mass of single diffusing molecules and nanoparticles. *Nat. Methods* **19**, 751–758 (2022).

26. G. Li, H. Su, G. Zheng, M. Zhou, W. Han, Y. Zhang, N. Ma, H. Wang, T. Klimach, Y. Cheng, Novel device for in situ and real-time detection of the acidity of ambient aerosols: Laboratory characterization and ambient measurements. *Environ. Sci. Technol.* **59**, 659–667 (2025).

27. H. Di, Z. Wang, D. Hua, Precise size distribution measurement of aerosol particles and fog droplets in the open atmosphere. *Opt. Express* **27**, A890–A908 (2019).

28. M. Meyer, F. Caruso, R. Lupoi, Particle velocity and dispersion of high Stokes number particles by PTV measurements inside a transparent supersonic Cold Spray nozzle. *Int. J. Multiphase Flow* **106**, 296–310 (2018).

29. B. van Heerden, N. A. Vickers, T. P. J. Krüger, S. B. Andersson, Real-time feedback-driven single-particle tracking: A survey and perspective. *Small* **18**, e2107024 (2022).

30. R. D. C. Moreira, J. Perchoux, Y. Zhao, C. Tronche, F. Jayat, T. Bosch, paper presented at the *2017 IEEE SENSORS*, Glasgow, Scotland, 29 October to 1 November 2017.

31. B. Hiller, R. K. Hanson, Simultaneous planar measurements of velocity and pressure fields in gas flows using laser-induced fluorescence. *Appl. Opt.* **27**, 33–48 (1988).

32. N. McDicken, A. Thomson, A. White, I. Toor, G. Gray, C. Moran, R. J. Watson, T. Anderson, 3D angle-independent Doppler and speckle tracking for the myocardium and blood flow. *Echo Res. Pract.* **6**, 105–114 (2019).

33. S. S. Ulyanov, Dynamics of speckles with a small number of scattering events: Specific features of manifestation of the Doppler effect. *Appl. Opt.* **53**, B94–B102 (2014).

34. R. Wang, W. Li, Z. Xia, H. Deng, Y. Zhang, R. Fu, S. Zhang, T. G. Euser, L. Yuan, N. Song, Y. Jiang, S. Xie, Optical trapping of mesoscale particles and atoms in hollow-core optical fibers: Principle and applications. *Light Sci. Appl.* **14**, 146 (2025).

35. R. Wang, K. Li, X. Liu, Y. Jiang, R. Yin, Y. Zheng, X. Jiang, S. Xie, Non-Markovian Doppler velocimetry of optically propelled microparticles in hollow-core photonic crystal fiber. *ACS Photonics* **11**, 1533–1539 (2024).

36. A. Sharma, S. Xie, R. Zeltner, P. S. J. Russell, On-the-fly particle metrology in hollow-core photonic crystal fibre. *Opt. Express* **27**, 34496–34504 (2019).

37. D. S. Bykov, O. A. Schmidt, T. G. Euser, P. S. J. Russell, Flying particle sensors in hollow-core photonic crystal fibre. *Nat. Photonics* **9**, 461–465 (2015).

38. M. K. Garbos, T. G. Euser, O. A. Schmidt, S. Unterkofler, P. S. J. Russell, Doppler velocimetry on microparticles trapped and propelled by laser light in liquid-filled photonic crystal fiber. *Opt. Lett.* **36**, 2020–2022 (2011).

39. O. A. Schmidt, T. G. Euser, P. S. Russell, Mode-based microparticle conveyor belt in air-filled hollow-core photonic crystal fiber. *Opt. Express* **21**, 29383–29391 (2013).

40. L. Fang, Z. Wan, A. Forbes, J. Wang, Vectorial Doppler metrology. *Nat. Commun.* **12**, 4186 (2021).

41. Y. Zhang, Z. Zhang, Q. Wang, Y. Zhao, High-accuracy transverse translation velocimeter enabled by OAM-assisted dual-point transverse Doppler effect. *APL Photonics* **8**, 096111 (2023).

42. Z. Wan, Z. Tang, J. Wang, Doppler effect tailoring: Extra-red shift of structured light. *Nat. Commun.* **16**, 10004 (2025).

43. Z. Huang, L. Cao, Quantitative phase imaging based on holography: Trends and new perspectives. *Light Sci. Appl.* **13**, 145 (2024).

44. Z. Mushtaq, M. Sharma, P. Bangotra, A. S. Gautam, S. Gautam, Atmospheric aerosols: Some highlights and highlighters, past to recent years. *Aerosol Sci. Eng.* **6**, 135–145 (2022).

45. Z. Wang, T. Liu, X. Yu, L. Kong, M. Huang, Ultra-high resolution particle size measurement based on scattering spectrum analysis—Simulation and experiment. *Opt. Express* **30**, 30480–30493 (2022).

46. I. R. Woodward, C. A. Fromen, Recent developments in aerosol pulmonary drug delivery: New technologies, new cargos, and new targets. *Annu. Rev. Biomed. Eng.* **26**, 307–330 (2024).

47. X. Feng, Y. Shi, Y. Zhang, F. Lei, R. Ren, X. Tang, Opportunities and challenges for inhalable nanomedicine formulations in respiratory diseases: A review. *Int. J. Nanomedicine* **19**, 1509–1538 (2024).

48. H. Cummins, N. Knable, Y. Yeh, Observation of diffusion broadening of Rayleigh scattered light. *Phys. Rev. Lett.* **12**, 150–153 (1964).

49. S. Sitar, V. Vezočnik, P. Maček, K. Kogej, D. Pahovnik, E. Žagar, Pitfalls in size characterization of soft particles by dynamic light scattering online coupled to asymmetrical flow field-flow fractionation. *Anal. Chem.* **89**, 11744–11752 (2017).

50. A. C. Makan, M. J. Spallek, M. du Toit, T. Klein, H. Pasch, Advanced analysis of polymer emulsions: Particle size and particle size distribution by field-flow fractionation and dynamic light scattering. *J. Chromatogr. A* **1442**, 94–106 (2016).

51. T. Dorfmüller, B. J. Berne, R. Pecora: Dynamic Light Scattering, John Wiley and Sons Ltd., Baffins Lane 1976, 376 Seiten. *Ber. Bunsen-Ges. Phys. Chem.* **81**, 101–101 (1977).

52. R. Finsy, Particle sizing by quasi-elastic light scattering. *Adv. Colloid Interface Sci.* **52**, 79–143 (1994).

53. A. Malloy, B. Carr, NanoParticle tracking analysis—The Halo™ system. *Part. Part. Syst. Char.* **23**, 197–204 (2006).

54. T. Wieduwilt, R. Förster, M. Nissen, J. Kobelke, M. A. Schmidt, Characterization of diffusing sub-10 nm nano-objects using single anti-resonant element optical fibers. *Nat. Commun.* **14**, 3247 (2023).

55. V. Filipe, A. Hawe, W. Jiskoot, Critical evaluation of Nanoparticle Tracking Analysis (NTA) by NanoSight for the measurement of nanoparticles and protein aggregates. *Pharm. Res.* **27**, 796–810 (2010).

56. P. Kramberger, M. Ciringer, A. Štrancar, M. Peterka, Evaluation of nanoparticle tracking analysis for total virus particle determination. *Virol. J.* **9**, 265 (2012).

57. A. D. Kashkanova, M. Blessing, A. Gemeinhardt, D. Soulat, V. Sandoghdar, Precision size and refractive index analysis of weakly scattering nanoparticles in polydispersions. *Nat. Methods* **19**, 586–593 (2022).

58. F. E. I. Li, H. Qi, C. You, Phase Doppler anemometry measurements and analysis of turbulence modulation in dilute gas–solid two-phase shear flows. *J. Fluid Mech.* **663**, 434–455 (2010).

59. X. Liu, W. H. Doub, C. Guo, Evaluation of droplet velocity and size from nasal spray devices using phase Doppler anemometry (PDA). *Int. J. Pharm.* **388**, 82–87 (2010).

60. J. Ma, S. C. M. Yu, H. W. Ng, Y. C. Lam, Some observations on particle size and velocity measurements using phase Doppler anemometry in plasma spray. *Plasma Chem. Plasma Process.* **24**, 85–115 (2004).

61. C. M. Jenkins, R. C. Ripley, C.-Y. Wu, Y. Horie, K. Powers, W. H. Wilson, Explosively driven particle fields imaged using a high speed framing camera and particle image velocimetry. *Int. J. Multiphase Flow* **51**, 73–86 (2013).

62. W. Wang, W. Xue, S. Wu, Z. Mu, J. Yi, A. J. Tang, High-speed micro-particle motion monitoring based on continuous single-frame multi-exposure technology. *Materials* **15**, 3871 (2022).

63. K. Balamonica, H. Goh Siang, Y. Toh Kai, Experimental study on the propagation of low-velocity impact waves in sand using particle image velocimetry. *Int. J. Geomech.* **20**, 04020209 (2020).

64. K. C. Lee, J. Guck, K. Goda, K. K. Tsia, Toward deep biophysical cytometry: Prospects and challenges. *Trends Biotechnol.* **39**, 1249–1262 (2021).

65. C. Gonzalez-Ballestero, M. Aspelmeyer, L. Novotny, R. Quidant, O. Romero-Isart, Levitodynamics: Levitation and control of microscopic objects in vacuum. *Science* **374**, eabg3027 (2021).

66. K. Li, R. Wang, S. Shao, F. Xie, Y. Jiang, S. Xie, Capture dynamics of dielectric microparticles in hollow-core-fiber-based optical traps. *Photonics* **10**, 1154 (2023).

67. P. Tortoli, G. Guidi, C. Atzeni, A review of experimental transverse Doppler studies. *IEEE Trans. Ultrason. Ferroelectr. Freq. Control* **41**, 84–89 (1994).
